# Factors in the Effective Use of Hearing Aids among Subjects with Age-Related Hearing Loss: A Systematic Review

**DOI:** 10.3390/jcm13144027

**Published:** 2024-07-10

**Authors:** Perrine Morvan, Johanna Buisson-Savin, Catherine Boiteux, Eric Bailly-Masson, Mareike Buhl, Hung Thai-Van

**Affiliations:** 1 Université Paris Cité, Institut Pasteur, AP-HP, Inserm, Fondation Pour l’Audition, Institut de l’Audition, IHU reConnect, F-75012 Paris, France; perrine.morvan@pasteur.fr (P.M.); mareike.buhl@pasteur.fr (M.B.); 2 Amplifon, 75014 Paris, France; johanna.savin@amplifon.com (J.B.-S.); catherine.boiteux@amplifon.com (C.B.); eric.baillymasson@amplifon.com (E.B.-M.); 3 Department of Audiology and Neurotology, Civil Hospitals of Lyon, 69003 Lyon, France; 4 Department of Physiology, Claude Bernard University, 69003 Lyon, France

**Keywords:** age-related hearing loss, presbycusis, hearing aids, patient satisfaction, signal processing, hearing aid fitting, personalized medicine, speech-in-noise

## Abstract

**Objectives:** Investigate factors contributing to the effective management of age-related hearing loss (ARHL) rehabilitation. **Methods**: A systematic review was conducted following PRISMA guidelines. The protocol was registered in PROSPERO (CRD42022374811). Articles were identified through systematic searches in the Scopus, PubMed, Web of Science, and Cochrane databases in May 2024. Only articles published between January 2005 and May 2024 were included. Studies were assessed for eligibility by two independent researchers and evaluated using the Crowe Critical Appraisal Tool v1.4 (CCAT). **Results**: Of the 278 articles identified, 54 were included. Three factors explain effective HA use. First, hearing aid signal processing, with directional microphones and noise reduction, improves user comfort and understanding regarding noise. Second, there is hearing aid fitting, with the NAL prescription rules as the gold standard, and bilateral, high-level HA performance for spatial localization and noise comprehension. Third, there is a patient-centered approach, using patient-related outcome measures (PROMs), questionnaires, counseling, and regular follow-up to involve patients in their therapeutic rehabilitation. **Conclusions**: Reaching a consensus on acoustic parameters is challenging due to variability in audiological results. Involving patients in their rehabilitation, addressing their needs and expectations, and offering individualized care are crucial.

## 1. Introduction

Age-related hearing loss (ARHL)—or presbycusis—is a physiological decline in hearing happening after the third decade of life. ARHL is bilateral, symmetric, progressive, and first affects high frequencies (HFs) [1]. Both intrinsic factors (genetic predisposition, epigenetic factors, biological aging) and extrinsic factors (noise exposure, ototoxic medications) play a role in the development of ARHL [2]. ARHL involves the degeneration of cochlear structures, including sensory hair cells, the stria vascularis, and spiral ganglion neurons. This degeneration is attributed to oxidative stress, mitochondrial dysfunction, DNA damage, and cellular senescence [3,4]. The proportion of the population affected by hearing loss (HL) rises gradually with age. A total of 13% of adults aged 50 to 59 versus 80% of subjects above 80 years old are diagnosed with ARHL [5].

Hearing-loss-related communication difficulties lead to social isolation [6,7,8,9], increased depression [5,8,10], and reduced quality of life [11]. Social factors, family factors, as well as the individual’s quality of life are all affected. Several studies have pointed out a possible link between self-reported [11,12] or measured ARHL [5] and the development of mild to severe neurocognitive impairments. The risk of incident all-cause dementia linearly increases with HL severity [6,7]. Hearing loss in mid-life may accelerate cognitive decline at later life stages [13,14].

Conversely, it has been shown, among large cohorts of ARHL subjects, that hearing aid (HA) use decreases the rates of cognitive decline and incident dementia [15,16,17]. The Lancet Commission on dementia prevention, intervention, and care has reported that the early fitting of HAs could benefit ARHL subjects, as 8% of cases of dementia could be prevented [18,19]. Some studies have also demonstrated the benefits of HAs regarding quality of life, disability, improved physical health, and cognitive decline [16,20].

As a result, access to HAs recently became a priority for national health agencies and authorities. The World Health Organization (WHO) [21] has released new technical guidance designed to provide practical assistance to countries developing hearing aid services in areas lacking human resources for assessing, fitting, and maintaining hearing aids. The Lancet [19] emphasizes the importance of encouraging hearing aid use to reduce the excess risk from hearing loss. The National Institute on Deafness and Other Communication Disorders (NIDCD) in the U.S. prioritizes improving hearing healthcare accessibility and affordability, collaborating with various agencies to address this urgent public health issue [22]. In this regard, it is crucial to assess the hearing status of each ARHL patient in detail to deliver a custom-hearing amplification program that best fits the patient’s needs. With identical pure tone audiometry thresholds, patients’ expectations and results may vary significantly from one subject to another. 

The benefits of HAs may depend on several criteria. For instance, signal-processing paradigms, which are central to HA functioning, have considerably progressed since the breakthrough in the late 1990s of digital technology in the development of HAs. Since then, auditory signal processing has been enhanced and adapted to better suit the patient’s hearing loss profile. Microphone directivity [23], signal compression [24], and hearing aid dynamics [25], among others, have been described as the three significant technical advances for signal processing in digital hearing aids. In 2005, more than 90% of hearing aids featured Digital Signal Processing technology, which is still widely used today [25,26]. 

In addition to digital signal processing (DSP) technology, the hearing aid fitting protocol is paramount for the success of ARHL rehabilitation [27]. Hearing care professionals (HCPs) deliver personalized hearing aid custom fitting based on embedded pre-determined amplification settings using HA benefit assessment tools. Such tools allow for functional gain measurements (i.e., aided versus unaided pure tone thresholds) as well as speech intelligibility evaluation in quiet and noisy backgrounds. They aim to quantify, in the best possible ecological conditions, speech recognition and discrimination changes that can be attributed to HA use [28]. 

However, hearing tests alone cannot fully reflect the benefits of HAs, the patients’ daily difficulties, and individual needs [29]. A wide range of questionnaires have been developed and validated to monitor factors such as satisfaction, utility, anxiety, depression, and quality of life. 

Self-administered questionnaires are simple, rapid, and practical tools for, on the one hand, getting ARHL patients actively involved in their hearing rehabilitation process and, on the other hand, assessing perceived benefits and progress in social integration thanks to HAs. Satisfaction may be independent of functional gain [29]. Self-assessment questionnaires have been developed since the 1990s, such as the Abbreviated Profile of Hearing Aid Benefit (APHAB) or Satisfaction Amplification in Daily Life (SADL) [30,31]. Other questionnaires, such as the Client Oriented Scale of Improvement (COSI) [32,33], appeared later to respond to the patients’ specific needs, i.e., expectations. 

Treating hearing-impaired (HI) people is, therefore, complex and can vary from one individual to another. To date, there is no real consensus on a single treatment plan or the tools that should be used to maximize the effectiveness and success of HA fitting. Several review studies have been conducted to determine which factors contribute the most to the effective use of HAs. The principal audiological determinants are the severity of HL [34,35] and the acceptance of background noise [35,36]. These papers recommend developing patient-reported measures and better training for healthcare professionals. These reviews, so far, have notable limitations, since none of them indicated the HL origin (e.g., sensorineural, mixed, or conductive) nor the age of the subjects. Furthermore, article inclusion criteria were often too broad, mixing studies from the 1980s, 1990s, and 2000s [34,35,36]. The latter do not represent current HA technology [25,26]. In addition, HI subjects’ needs and expectations have grown in parallel with technology and the increasing early use of HAs. Nowadays, a wide range of HAs can be fitted depending on the patient’s age and other audiological, morphological, aesthetic, or praxis-related factors. 

Given the multiple consequences of presbycusis for the lives of the elderly and the variety of HA benefit assessment tools, investigating the factors contributing to the effective management of ARHL rehabilitation and finding a common strategy are crucial. We have defined “effective use” as the factors contributing to the successful adoption and continued use of HAs among HI people. This concept encompasses various factors that enhance the likelihood of individuals not only adopting but persistently using hearing aids. The purpose of this systematic review is to identify precisely which factors contribute to the effective use (O) of hearing aids (I) among ARHL subjects (P). 

## 2. Methods

### 2.1. Search Strategy

The Preferred Reporting Items for Systematic Reviews and Meta-Analyses (PRISMA) guidelines [37] (Table S1) were used for the review design and methodology. The protocol was registered in the PROSPERO International Prospective Register of Systematic Reviews on 25 November 2022 (CRD42022374811). Research was conducted between November 2022 and May 2024 using the Scopus, PubMed, Web of Science, and Cochrane databases. Keywords for searches in the various databases were defined using the PICO method [38]. The databases were searched using the Mesh descriptors “age-related hearing loss”, “presbycusis”, “hearing aids”, “patient satisfaction”, “audiological factors”, and all their synonyms (Table S2). To ensure that the signal processing present in HAs is still commonly used, only articles published since January 2005 were included in the second selection stage. Indeed, since the advent of digital hearing aids, three major advancements are notable: the transition from simple to adaptive directionality [39], the shift from fixed to adaptive compression [40], and the expansion of the dynamic range [41]. In 2005, 93% of the hearing aids sold in the United States contained DSP technology [25,26], a technology still used in the current hearing aids, making comparisons easier. 

No recent systematic reviews on the same topic were identified in the Cochrane Library. The articles needed to include presbycusis subjects (bilateral sensorineural loss) aged 18 and older and wearing conventional HAs bilaterally or unilaterally. For each article, we ensured that the inclusion criteria or results indicated the following denominations: “bilateral/sloping/symmetrical sensorineural hearing loss/hearing impairment”, “pure-tone air-conduction hearing thresholds consistent with age-related hearing loss”, and “diagnosed with presbycusis”. When these were not available [42,43,44,45], a figure corresponding to the average audiogram of the subjects was available and compared to the criteria of ARHL (Table S3, Column 5). For four articles [46,47,48,49], these two parameters were not available. Therefore, the assumption of ARHL was only based on the age of the subjects, which was 73.4 ± 9.8 years in these studies. Subjective and objective evaluation methods were selected. In the case of subjective methods, the validation of the questionnaires was ensured. We also included studies examining audiological factors’ short- and long-term effects on HI people. Regarding the study design, randomized controlled trials, non-randomized controlled interventions, and observational studies were included. The methodology had to be clearly explained. Psychometric and functional scales had to be valid. Because of the heterogeneity of outcomes, a meta-analysis could not be performed. 

Articles involving minors (defined later as “different population”), non-hearing communication disorders, cognitive deficits, congenital syndromes, Meniere’s disease, tinnitus, and other comorbidities were excluded. These criteria were assessed and excluded based on the inclusion criteria specified in the articles. Assistive listening devices, cochlear implants, or bone-anchored devices were also excluded, as were conductive, retro-cochlear, or noise-induced hearing losses (defined later as “different outcome”). We also excluded systematic reviews and meta-analyses (defined later as “different publication criteria”). Articles written in languages other than English were not included. 

### 2.2. Data Extraction 

Two reviewers analyzed each article using Rayyan software [50]. A third reviewer managed conflict. The search identified 278 articles, which were imported into the Rayyan software. First, 104 duplicates from different search engines were eliminated. After reading the titles and abstracts, an initial selection was made. The complete articles were then evaluated according to inclusion and exclusion criteria. 

### 2.3. Quality Assessment 

All the chosen studies thoroughly assessed methodological quality, revealing a consistently high standard of evidence. Due to the heterogeneity of the studies (both experimental and observational), it was necessary to find a tool that could evaluate the full diversity of the research. The Crowe Critical Appraisal Tool (CCAT), developed by Crowe and Sheppard in 2011 [51,52], was therefore used as the instrument for quality assessment in this review (Figure S1). The CCAT is a single tool for appraising all studies, regardless of their research design, to enable direct comparison and enhance conclusions and recommendations. Widely applicable to various study designs in health research, the CCAT stands out as one of the few critical appraisal instruments that have undergone rigorous evaluation for validity and reliability [52,53]. This tool enables the evaluation of studies across eight key categories: Preamble, Introduction, Design, Sampling, Data Collection, Ethical Matters, Results, and Discussion. Multiple items are scrutinized within each category, and a six-point scale (ranging from 0 to 5) is employed for scoring. A category is assigned a score of 0 if there is no supporting evidence in the study, 1 if there is minimal evidence, and 5 if there is substantial and sufficient evidence. 

### 2.4. Statistical Analysis 

Due to the heterogeneity of the results, we could not carry out a meta-analysis of all the articles. Consequently, we have selected one direction for this analysis. Hearing loss affects audibility and perceptual sound quality [54,55]. People with ARHL often report difficulties in understanding speech in noise. We conducted a meta-analysis of speech-in-noise studies to provide a more comprehensive description and visualization of hearing aid-related factors that may influence the speech reception threshold (SRT) in hearing aid users with ARHL. Wherever possible, we have recorded the statistics as they appear in the original articles to ensure validity and reliability. We used graphical reading to extract the data when the results were presented graphically. 

## 3. Results 

### 3.1. Data Extraction 

**Figure 1:**
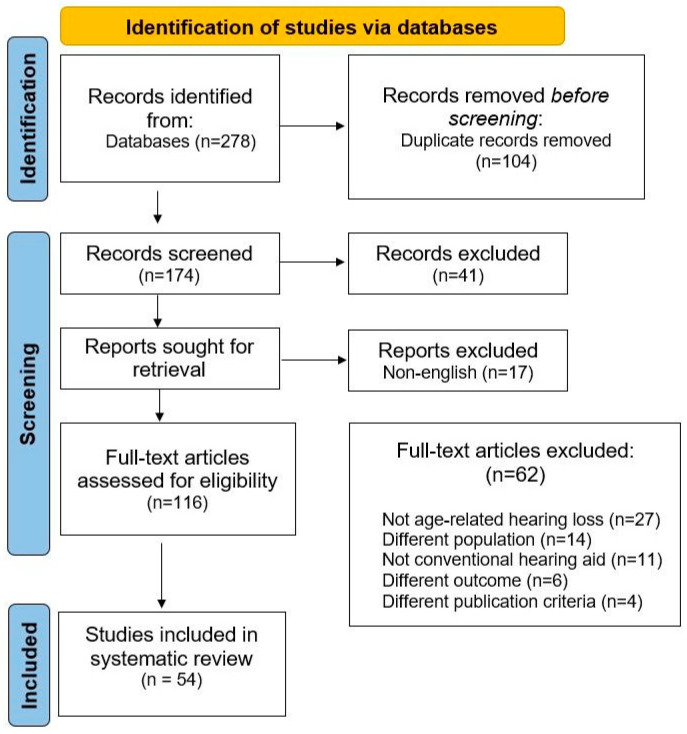
PRISMA 2020 flow chart diagram for updated systematic reviews which included searches of databases and study selection. Last date of search is May 2024.

Our search of the four databases identified 278 articles (Figure 1). After eliminating duplicates, an initial selection based on reading the titles and abstracts excluded 41 articles. Seventeen papers not written in English were removed. After completing the remaining 116 articles, 62 additional studies were excluded. In the end, 54 studies were included in our systematic review, and their characteristics are listed in Table S3. 

### 3.2. Study Characteristics 

Of the 54 articles included in our review, the majority were experimental studies, including 21 crossover studies [23,42,56,57,58,59,60,61,62,63,64,65,66,67,68,69,70,71,72,73,74], 10 randomized crossover studies [75,76,77,78,79,80,81,82,83,84], 5 randomized controlled trials [85,86,87,88,89], 3 non-randomized controlled trials [90,91,92], and 1 parallel group [45]. Fourteen observational studies completed the analysis, including seven cross-sectional studies [44,47,48,85,93,94,95], three longitudinal studies [96,97,98], two cohort studies [46,99], and two retrospective studies [43,49] (Table S3, column 2). Twenty-six studies were conducted in America, fourteen were conducted in Europe, seven were conducted in Oceania, and seven were conducted in Asia. The participants ranged in age from 18 to 95 years (mean = 69 ± 7.2 years), with 79.5% over 65 years. The sample sizes ranged from 8 [23] to 1464 [49] participants. In these studies, men were more frequently recruited (55.8%) than women (44.1%). The review included nearly the same number of new hearing aid users (21 articles) as experienced users (18 articles). Additionally, ten studies included both conditions, while five studies did not provide this information [42,45,49,74,92]. 

### 3.3. Quality Appraisal 

The articles included in the review were assessed using Crowe’s critical appraisal tool. According to this scale, the level of evidence could be classified between 0 and 40. All the articles in the review had a score between 23 and 35 (mean = 29), which indicated that these studies had a reasonably good level of quality (Table S4). The studies included in the review explored various potential factors that could explain the effective use of hearing aids among ARHL adults. Three principal factors were identified in the review: signal processing, which was examined in 22 articles; hearing aid fitting, covered in 42 articles; and patient experience, explored in 44 articles. 

### 3.4. Factor 1: Signal Processing 

Hearing aid signal processing is the focus of this study. In particular, dynamic compression, frequency processing, microphone directionality, noise reduction, the number of processing channels, and anti-feedback are the most commonly used features to determine the ideal set of signal processing in hearing rehabilitation using HAs. 

#### 3.4.1. Dynamic Compression

Seven of the included papers studied compression. Overall, the linear settings Channel Free and Wide Dynamic Range Compression (WDRC) were used, but WDRC was the most widely studied [66,67,68,76,80,81,88]. This compression mode translates the dynamics of normal-hearing people into those of HI people. Low-intensity sounds are more strongly amplified than in linear settings. Compared to linear settings, comfort, satisfaction, and intelligibility are superior when using WDRC, which seems more appropriate for presbycusis patients in challenging backgrounds [80]. However, linear settings seem more appropriate for subjects with slight HL, reduced auditory dynamics, and quiet everyday situations [81]. Nonetheless, the results are unclear when WDRC compression is compared with other signal processing systems, such as Channel Free [67,68]. Indeed, both types of signal processing give equivalent results in terms of speech perception and consonant identification in quiet backgrounds as in noisy backgrounds [68]. The patient satisfaction with both strategies is equivalent [67]. 

#### 3.4.2. Frequency Processing 

Other parameters were investigated, such as the use of frequency transposition. No additional advantage over conventional gain had yet been demonstrated [88]. Slow [62] and fast [66] compression yields different results. While the first option, coupled explicitly in this study to a CAMEQ2-HF preset providing a more substantial gain above 7500 Hz [62], is perceived as uncomfortable and somewhat noisy, the second does not reduce satisfaction compared to the more conventional use of WDRC. Another sound-processing strategy used in HAs is non-linear frequency compression (NLFC). It is supposed to improve the audibility of HF sounds. As reported in the article by Chen et al. [79], NLFC helps to enhance the recognition of sentences and fricative sounds when activated. In addition, compared with NLFC compression alone [69], the combined use of directional microphones and NLFC compression improves satisfaction. As the subject perceptions can considerably vary when using one specific signal processing compared to another, self-reported measures are important in documenting the perceived responses of HA users [76]. 

#### 3.4.3. Microphone Directionality 

Microphones’ directional characteristics were another critical element in HA processing, addressed in four studies. Fixed and adaptive directional microphones both significantly improve speech-in-noise intelligibility [23,45]. Wu et al. [91] report that the contribution of directionality is more limited in older subjects (72.3 ± 3.6 years) than in younger subjects (55.4 ± 8.9 years). Indeed, it may be detrimental to elderly subjects’ intelligibility, since they are, on average, less exposed to challenging backgrounds. However, Neher et al. [84] show that severely HI subjects benefit from directional microphones. 

#### 3.4.4. Noise Reduction 

Since the breakthrough of digital HAs, the analysis of different everyday situations in real time has continued to improve. As HI subjects’ hearing dynamics and signal perception differed from those of normal-hearing subjects, many noise reducer (NR) algorithms were developed. However, speech intelligibility must not be deteriorated by the activation of these reducers. Korhonen et al. claim that using transient noise reduction [59] or wind noise attenuation [60] does not impair speech comprehension and consonant recognition. There is no perceived difference between different intensities of digital noise reducers (DNR) [77]. However, the use of different types of noise reducers like DNR [70,72,77] and transient or wind NR [59,60] reduce discomfort. However, patients’ expectations in challenging backgrounds are often unrealistic. It is therefore essential to educate the patients on the goals and expectations they should have with their HA on a daily basis [90]. 

#### 3.4.5. Number of Processing Channels 

Other acoustic parameters were also studied. In the study of auditory dynamics by Mispagel et al., an increase in signal processing channels from 32 to 64 did not seem to affect comfort, satisfaction, speech comprehension in quiet or in noise, nor the subject’s perception of speech [61]. 

#### 3.4.6. Anti-Feedback 

Another well-known phenomenon was feedback from hearing aids. This occurs when the acoustic signal escapes from the ear canal and reaches the microphone in the hearing aid. Only one study reports on anti-feedback systems [42]. It does not significantly degrade music quality or intelligibility. 

### 3.5. Factor 2: Hearing Aid Fitting 

The second part of this study focused on hearing aid fitting. In particular, we noted that hearing aid fitting methods, functional gain assessment, communication aids, unilateral and bilateral fittings, and hearing aid performance levels were mainly studied in the review. 

#### 3.5.1. Hearing Aid Fitting Methods 

Most HAs were programmed using a National Acoustical Laboratories (NAL) prescription [100]. Fifteen studies used the NAL-NL1 methodology [42,46,59,66,67,68,75,77,83,85,86,88,90,94,99,101], eleven used the more recent NAL-NL2 version [57,70,71,72,73,76,78,82,87,88,96,102], and three used the NAL-RP methodology [80,81,95,103]. Four studies [63,65,69,78] based their parameters on the Desired Sensation Level (DSL) v5 or DSL io methodology [104]. Finally, six studies [56,61,79,84,89,91] used the manufacturer’s methodologies embedded in their own HAs. Only two studies specifically evaluated the contribution of one methodology versus the other [75,78]. The results show that NAL-type methodologies are often more appreciated than manufacturers’ methodologies [75]. DSL v5 is more efficient than NAL-NL2 [78] in quiet backgrounds. 

#### 3.5.2. Functional Gain Assessment 

While most studies had used questionnaires, they were not as thorough with intelligibility benefit assessment. The most commonly used tests in noise were the Hearing-in-Noise Test [23,57,61,65,67,71,72,78,79,86,91,105] and the Connected Speech Test [65,77,87,91,106]. Wu et al. [73] used the Four Alternative Auditory Feature Test. For speech-in-noise tests, Korhonen et al. [59,60] used the Office of Research in Clinical Amplification Nonsense Syllable Test [107] and Plyler et al. [67,69] used the Pascoe’s High-Frequency Word List [108]. Munro et al. [63] used the Bamford Kowal Bench [109], a test designed to assess intelligibility in quiet and noise. Three studies [57,65,69] used the Acceptable Noise Level [110] test to assess subjects’ central ability to follow a speech source without being disturbed by another noise source. 

#### 3.5.3. Communication Aids 

Only a few studies used assistive listening devices. The evaluated features were the use of the HA’s volume by a control button [56,61,80] or remote control [82] as the implementation of several specific listening programs [23,56,61,65,71,77]. Wu et al. implemented a smartphone application in two studies [72,73]. Solheim et al. [48] also added the T-coil. In these studies, the patients accept the basic settings in 75% of situations [56]. In certain specific conditions and depending on the patient’s needs [48], it is recommended to create a second personalized program [56,71] or to set up a volume control [56] to improve compliance [48]. Everyday speech-in-noise situations can be managed by automatic speech recognition software [70]. 

#### 3.5.4. Unilateral and Bilateral Fittings

Two studies compared bilateral and unilateral fitting. Patients fitted bilaterally achieve better results in spatial localization, sound detection, and speech in quiet. However, they tend to be more bothered by amplification and experience less comfort [43,93]. Patients are more likely to be satisfied with their HAs if needs and expectations have been previously identified. This must be considered in the HA fitting process and patients’ expectations must be designed to meet realistic goals [93]. 

#### 3.5.5. The Performance Level of Hearing Aids 

Patients fitted with high-performing HAs achieve better results in terms of speech-in-noise intelligibility and spatial localization than those fitted with more basic HAs [43,57,72]. Patients with moderate HL are also more likely to benefit from high-performing HAs [72]. Although acoustic parameters were crucial for a successful HA fitting, it became increasingly important to consider the patient’s perception. To this end, several studies attempted to identify factors likely to improve HA use in older people with HL. For this, the utilization of standardized questionnaires, specific training programs, patient-centered care, and patient self-adjustment were studied. 

### 3.6. Factor 3: Patients’ Experience 

#### 3.6.1. Standardized Questionnaires 

Most studies (N = 41/54, 75%) used questionnaires. The main ones were benefit and satisfaction questionnaires, with 48 different standardized and 7 non-validated questionnaires [23,56,57,66,67,68,95]. The most commonly used satisfaction questionnaires were the APHAB [30] used in 11 studies [44,62,65,67,72,73,75,77,80,88,90] and in Boymans’ study [43] only with the “Aversion to Sounds” subscale; the International Outcome Inventory for Hearing Aids [111] was carried out in 9 studies [43,83,85,86,89,90,93,99,112]; the Satisfaction with Amplification in Daily Life [31] was performed in another 8 studies [44,46,72,73,80,85,97,112]; and the Glasgow Hearing Aid Benefit Profile [113] was run in 4 studies [45,46,73,80]. Other questionnaires—mainly the Handicap Inventory for the Elderly [49,64,73,83,85,87,96,112,114] but also the Practical Hearing Aid Skills Test [44]—were used to characterize patients’ discomfort and handicap. Quality of life was mainly assessed using the Speech, Spatial, and Qualities of Hearing Scale [115] in six studies [23,57,71,72,73,76] and the Sense of Coherence Scale in two studies [85,112,116]. Depression was also assessed using the Geriatric Depression Scale [64,77,117] and the Hospital Anxiety and Depression Scale [85,112,118]. On the other hand, the assessment of HA sound quality was quantified by a single standardized questionnaire, the Profile of Aided Loudness [119] in the study by Moore et al. [62]. Other studies on this parameter created evaluation scales [61,62,72,79]. Finally, other criteria were taken into consideration, such as communication strategies with the Communication Strategies Scale questionnaire [120] in the articles by Öberg et al. [64,85,112] or hearing loss-related disability with the Hearing Handicap and Disability Inventory questionnaire [121] in the article by Boymans et al. [43]. The COSI questionnaire [32,33], which questions the patients’ specific complaints, was used in five studies [64,77,85,89,96]. 

#### 3.6.2. Specific Training Programs 

Some studies suggested that the device adjustment period should be combined with specific training to improve patient compliance. Although these training programs are, for example, interactive weekly sessions for people with HL and their significant others to discuss communication difficulties and generate communication solutions, they also permit participants to make simple adjustments to create their settings thanks to listening scenarios on a computer or directly on their hearing aids. These programs do not show any significant improvement in patient compliance. However, patients perceive some benefits in increasing their ability to deal with their HL and the problems it creates [64,82,83,96]. It is also easier for audiologists to incorporate a follow-up into their clinical routine [95,96]. These training programs benefit subjects with previous fitting experience rather than first-time users [83]. Furthermore, preparing new users with a gentle preset designed to gradually acclimate them to HAs is no more beneficial than a conventional fitting follow-up [85].

#### 3.6.3. Patient-Centered Care 

Handling the HA is often seen as a barrier to fitting [44,48]. Pain associated with earmolds can also contribute to the non-use of HAs, as can the perceived discomfort of the amplification [48]. To limit these downsides, it seems necessary to consider the patients’ perception during the fitting and follow-up [47,48,89,97,112]. A practical solution for healthcare professionals and patients may be using questionnaires before, during, and after fitting [44,97,112]. As this follow-up is sometimes difficult or even impossible due to the patient’s lack of mobility, the patient’s geographical location, or the absence of nearby professionals, teleconsultation appears to be an effective service model for programming and monitoring HAs, as well as for providing informative advice for those specific cases [86]. The assessment of quality of life [98] or personality [99] can also help the clinician to better manage the patient. People with more extroverted personalities and a more significant perceived disability have better outcomes [99]. HA fitting can also improve the overall quality of life. However, it is necessary to also develop programs to support the reintegration of elderly subjects into society after long periods of social isolation [98]. To overcome some disadvantages of retrospective questionnaires, administered at a single point in time, Shiffman et al. created the Ecological Momentary Assessment (EMA) methodology [122]. Respondents have to report several times on their experiences during or shortly after the experiments, such as in situ self-evaluations. This new data collection method can complement the already-in-use questionnaires and provide additional information on defined HA parameters and patient needs [73]. 

#### 3.6.4. Patient Self-Adjustment 

With the development of smartphone applications, patients became increasingly involved with their HAs. There were also alternative HAs, known as over-the-counter (OTC) HAs. OTCs were purchased and paid for directly by the patient without going through an audiologist. Humes et al. [87] compare these alternative devices with conventional HAs. The results of OTC device are inferior to those obtained with conventional HAs. However, they find that patients who purchase conventional HAs are more likely to reject them. Cho et al. [49] further identify that third-party reimbursement is the most significant factor influencing the intention to purchase HAs. In addition, Ferguson et al. [46] find no convincing results for implementing HA self-assessment. They also add that the use of the Measure of Audiologic Rehabilitation Self-efficacy for Hearing Aids questionnaire is not appropriate for this type of assessment. Nevertheless, it is essential to consider the patient’s needs in the clinical assessment. When patients are in control of their amplification, the main difference between patients lies in high-frequency (HF) adjustments. While the high frequencies undoubtedly provide optimal speech clarity and improved speech-in-noise performance [63,65,94], patients often tend to reduce them in order to achieve greater comfort [58,63]. 

### 3.7. Speech Reception Threshold and Satisfaction 

Among the 54 articles in the review, 20 incorporated speech intelligibility tests administered in quiet environments, noisy settings, or both. Notably, 14 studies employed speech-in-noise tests, while 11 utilized the Hearing-in-Noise Test (HINT). As highlighted in the preceding section, patient satisfaction is imperative for enhancing management practices. Hence, we targeted studies that concurrently addressed speech test outcomes and patient satisfaction parameters. Ultimately, our dataset comprised eight articles. Each Speech Reception Threshold (SRT) value underwent normalization to facilitate meaningful analyses, aligning with the normative HINT values validated in the respective language employed (Brazilian [123], American [124], and Mandarin [125]). Furthermore, subjective satisfaction measures were uniformly normalized on an invert scale ranging from 0 (unsatisfied) to 100 (absolutely satisfied) despite variations in the specific tests administered across studies. Data are available in Table S5. 

**Figure 2:**
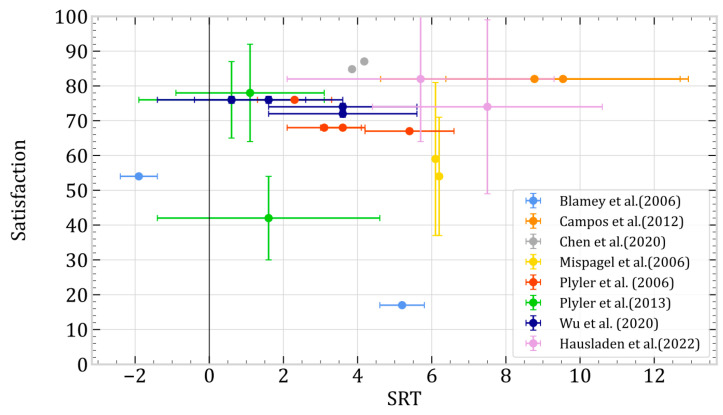
Speech Reception Threshold in Noise and Satisfaction depending on each author [23,57,61,65,67,73,79,86].

**Figure 3:**
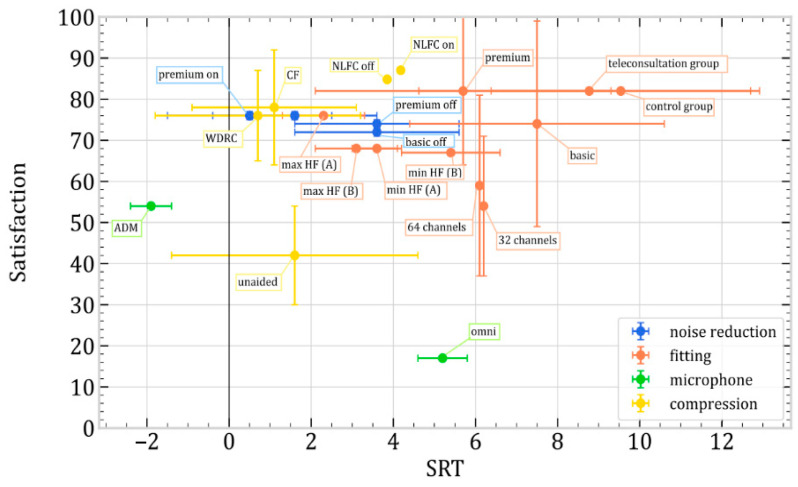
Speech in noise and satisfaction depending on various signal processing and fitting factors.

Figure 2 shows the results depending on each article. For better identification, the articles were categorized based on the sub-factors of signal processing and fitting investigated for streamlined analysis (Figure 3). Four sub-factors were identified: noise reduction algorithms, hearing aid settings, microphone use, and different types of compression.

The application of NLFC compression achieves optimal satisfaction scores [79]. However, when NLFC is deactivated, comprehension in noisy conditions surpasses that when activated. It is important to note that the unavailability of standard deviation information prevents the determination of statistical significance for these data. The article specifies the absence of statistically significant results in this regard. In noisy environments, using an adaptive microphone (ADM) significantly improves comprehension, producing scores that exceed the normative value. In comparison to the utilization of omnidirectional microphones, both satisfaction and comprehension in noise demonstrate significant improvement [23]. Utilizing WDRC or CF compression, enabling noise reducers, and optimizing high-frequency settings all contribute to achieving a favorable satisfaction-to-understanding ratio in noisy environments [65,67,73]. Conversely, deactivating noise reducers and minimizing high-frequency amplification yield inferior outcomes in both noise and satisfaction. Enhancing the number of adjustment channels, using premium hearing aids, and fine-tuning them through teleconsultation led to improved results in understanding speech in noisy conditions [57,61,86]. However, these parameters are only some of the critical factors for optimizing comprehension in noisy environments. 

## 4. Discussion 

This systematic review aimed to identify the factors contributing to the effective use of hearing aids among ARHL subjects. We identified three main factors: signal processing, hearing aid fitting, and the patient-centered approach. 

### 4.1. Signal Processing 

The two most widely used and studied compressions in our review are WDRC and linear compression. WDRC compression gives better results regarding comfort, satisfaction, and intelligibility and is more suitable for active-in-daily-life ARHL subjects [80]. Linear compression, on the other hand, is best suited for subjects with slight hearing loss, reduced auditory dynamics, and relatively quiet lifestyles [81]. While several studies of the literature suggest that WDRC performs better for intelligibility in quiet and hearing comfort [126,127], others suggest that linear compression is better for intelligibility in quiet and noisy backgrounds and for speech quality [128,129,130]. Audiologists can leverage these findings in clinical practice by tailoring the compression algorithm choice based on the patient’s lifestyle and hearing needs. For active patients, WDRC is recommended to enhance speech clarity and comfort in various environments, while linear compression is ideal for those leading quieter lives, ensuring a natural listening experience without excessive amplification. 

The consequences of using fixed and adaptive directional microphones were investigated. While some authors recommend activating directional microphones for younger subjects who are more likely to be confronted with complex and noisy situations in their daily lives [91], others recommend them for subjects with more significant HL to provide them with maximum comfort [84]. Activating adaptive directional microphones for younger patients can enhance their ability to focus on conversations in crowded settings, such as restaurants or social gatherings. 

Using noise reduction algorithms does not affect speech understanding or consonant recognition [59,60]. Other authors suggest that noise reduction algorithms improve comfort and reduce the listening effort [131,132,133]. However, they do not find any improvement in comprehension in noise. High noise reduction increases the risk of information loss and negative gains. In addition, they are only effective in the case of stable, highly impulsive noise and less so in the case of “cocktail party”-type noise. Furthermore, not all noise reduction algorithms have the same properties. Activation times and operating modes vary from one manufacturer to another. The combined activation of noise reduction and directional microphones consistently improves HA user comfort and understanding in noise [59,60,70,72,77]. Johnson also studies the feedback-canceller impact on speech comprehension performances and music listening [42] and finds none. This result seems surprising for music listening, as numerous studies show that using feedback-cancelling can reduce HF amplification [41,134], produce artifacts, and alter the quality of periodic sound [135,136]. In addition, while open earmolds are preferred and more readily accepted by patients due to their comfort, signal processing efficiency and signal-to-noise ratio improvement are typically achieved with closed or lightly vented earmolds [137]. This review found that most studies do not adequately consider this parameter, which is critical for the effectiveness of the chosen signal processing. Clinicians should carefully weigh the trade-offs between comfort and performance when recommending earmold types, especially for patients needing improved signal processing efficiency. 

Moreover, significant individual differences in the venting effect (VE) and insertion gain across different ear tips have been highlighted, emphasizing the importance of personalized fitting [138]. Real ear verification is essential in clinical practice to ensure that the prescribed gain is achieved at the eardrum, thus preventing individual differences in VE from affecting fitting outcomes, particularly at low frequencies. This approach ensures a stable and effective fitting over time, tailored to the unique acoustic properties of each patient’s ear canal. While technology development has undoubtedly improved user comfort, it is not easy to reach a consensus on using acoustic parameters to optimize the effective use of HAs. 

### 4.2. Hearing aid Fitting 

#### 4.2.1. Prescription Formulas 

The most widely used prescription formulas for HAs assessment are the NAL-NL1 and NAL-NL2 prescription rules. Whereas they are often considered more comfortable than manufacturers’ methods by HI subjects [75], they remain less efficient for speech-in-quiet intelligibility than a DSL-v5-type formula [78]. NAL formulas evolve through time based on numerous studies and statistical analyses of fitted HAs patients’ results. They were developed based on HAs features more than 20 years ago [101,102,103,139]. By 2005 [140], NAL methods became the prescription formula gold standard. They are still widely used today, even though HA technology has considerably evolved. Future studies should use prescription rules based on both liminal and supraliminal thresholds to consider the patient’s hearing dynamics and to select the optimal real-ear output of hearing aids [140]. In addition, these prescriptions should be supplemented by the ear’s natural response without a hearing aid to adjust the prescription to the natural amplification of the patient’s ear. If the fitting does not meet the patient’s needs, adjustments in gain and output may be necessary to ensure optimal comfort and performance [141,142]. 

#### 4.2.2. Bilateral vs. Unilateral Fitting 

With a given hearing loss profile, bilateral HAs fittings give better results than unilateral HAs for spatial localization, sound detection, and speech-in-quiet intelligibility [43,93]. Higher-performing HAs also allow users to achieve better results for speech-in-noise intelligibility and spatial localization [43,93]. These results are generally consistent with the literature [143,144,145]. When in 75% of cases, the basic HA setting is considered effective [56], adding additional programs or volume control is appropriate in specific situations, depending on the needs and expectations of the patient [48,56,71]. The amplification self-adjustment should be supervised since patients will tend to reduce HFs amplification to gain comfort, which impairs speech-in-noise intelligibility [58,63,65]. Whenever possible, audiologists should recommend bilateral fittings for patients to improve spatial localization and sound detection. For patients with active lifestyles or those frequently in noisy environments, high-performing hearing aids with advanced signal processing should be recommended. Implementing custom programs for different listening environments, such as quiet homes and noisy restaurants, can enhance the user’s experience and satisfaction [146,147]. 

### 4.3. Patient-Centered Approach 

While it is difficult to reach a consensus on signal processing and hearing aid fitting, papers are quite unanimous on the use of the patient-centered approach. As mentioned in most of the selected studies, patients’ needs must be considered from the start in HA fitting and during regular long-term follow-ups. As needs may change over time, using questionnaires, counseling, and regular follow-up seems essential to involve patients in their therapeutic rehabilitation [44,47,48,97,112]. These findings highlight the value of using a patient-related outcome measures (PROMs) approach that has been tested in physiotherapy for many years [113,148,149,150]. It consists of directly involving the patient by defining personalized goals based on the patient’s priority needs. Goal Attainment Scaling is a method for creating personalized assessment scales to quantify the achievement of rehabilitation goals. It is mainly used in cognitive rehabilitation medicine [148] but can apply to all types of rehabilitation, particularly neurosensory rehabilitation. In audiology, PROMS approach is emerging [151,152,153]. Healthcare professionals should use PROMs to involve patients in their rehabilitation, setting personalized goals and monitoring their progress. Scheduling regular follow-up appointments is important in reassessing patient needs, adjusting settings, and providing ongoing support. The conclusion of these articles is unanimous: It is now necessary to co-construct the axes of rehabilitation with the patient to optimize therapeutic adherence.

### 4.4. Speech Reception Threshold and Satisfaction 

Finally, due to the heterogeneity of the results, we focused our analysis on factors influencing the speech reception threshold (SRT) in hearing aid users with ARHL. Activating NLFC compression achieves optimal satisfaction scores, though comprehension in noisy environments is better without it. Using an adaptive microphone significantly improves comprehension in noise. Additionally, enabling WDRC or CF compression and noise reducers and optimizing high-frequency settings contribute to better satisfaction and understanding in noisy conditions. 

### 4.5. Limits 

Our study has several limitations. First, most of our articles recruited fewer than thirty participants. Overall, the sample sizes are too small to draw general conclusions. In addition, we excluded systematic reviews, meta-analyses, and articles written in a language other than English. Publication and language biases are to be expected. By excluding systematic reviews and meta-analyses, we may have overlooked comprehensive evaluations of existing literature that could provide a more balanced view of the findings. Including only English-language articles further exacerbates this issue, as important studies published in other languages are not considered, which may lead to incomplete or biased interpretations of the data. This exclusion limits the diversity of the data and may result in a bias towards findings prevalent in English-speaking countries, potentially missing cultural or regional variations in study outcomes. 

Additionally, the heterogeneity among the studies, such as the use of different questionnaires and varied methodological approaches, further complicates the synthesis of the results. The wide variety of instruments used by different authors introduces variability that challenges the consistency and comparability of findings across studies. The lack of standardized outcome measures and the reliance on diverse, often non-validated instruments hamper the ability to perform a meta-analysis. Consequently, our review provides mostly qualitative generalizations rather than quantitative syntheses of the data. In addition, one of our inclusion criteria was to include studies with adult subjects (aged 18 and older). However, we find that the patient profile is essentially the same, i.e., subjects with moderate HL. Taking into consideration the degree of HL would be interesting to stratify the results according to the different populations. Furthermore, all conclusions of this review are only available for adults and presbycusis people, the most common population seen by audiologists in a daily routine. It is only possible to make suggestions for this part of the population. It could be interesting to continue this review for other types of HL, like conductive or mixed, because the approach by an audiologist is completely different. Also, the determination of the origin of HL depends only on results at audiometry thresholds via headphones. It is therefore impossible to conclude whether the HL is acquired or not. 

### 4.6. Future Research 

Future research is needed to extend the literature on the effective use of HAs. First, it would be interesting to evaluate the effect of one signal treatment feature (compression, noise reduction, directionality, etc.) over another. This would help in better understanding how HAs operate and enable HCPs to use all available treatments effectively. It is also necessary for the studies to use modern pre-setting methods using liminal and supraliminal thresholds. This would ensure that the drawn conclusions fully account for the technical characteristics of current HAs. In addition, different acoustic parameters could be systematically studied at different degrees of HL. Clinical trials should also generalize the PROMS methodology. For example, an innovative approach to adapting HA could be explored. The amplification of HA could be based not on thresholds but on the results of speech and supraliminal tests to better meet patients’ objectives, which are mainly related to their comprehension problems in a noisy environment. Finally, it would be more relevant to conduct these studies on larger patient panels to increase the statistical power of the results and perform a meta-analysis if data were available. 

## 5. Conclusions

This review aimed to identify the factors contributing to the effective use of HAs among ARHL subjects. Given the wide variability in audiological results, reaching a consensus on using specific acoustic parameters takes work. Nevertheless, it seems necessary to involve the patients, to listen to their specific needs, and to offer them individualized care throughout their therapeutic rehabilitation. However, future studies must use modern signal processing techniques, especially in the equipment pre-setting methodology. It would also be interesting to systematically study the benefit of different signal processing and fitting characteristics in relation to the patients’ needs to potentially derive links between patient profiles and hearing aid fitting properties. 

## Data Availability

Data is contained within the article or Supplementary Materials.

## Supplementary Materials

The following supporting information can be downloaded at: https://www.mdpi.com/article/10.3390/jcm13144027/s1, Figure S1: CCAT form; Table S1: PRISMA Checklist [154]; Table S2: Search Terms; Table S3: Study Characteristics and CCAT Scores; Table S4: CCAT Scores; Table S5: Table of Normalized SRT Values and Subjective Satisfaction Scores Across Studies.

## Abbreviations

The following abbreviations are used in this manuscript:

APHAB	Abbreviated Profile of Hearing Aid Benefit
ARHL	Age-Related Hearing Loss
CCAT	Crowe Critical Appraisal Tool
COSI	Client-Oriented Scale of Improvement
DNR	Digital Noise Reducers
DSL	Desired Sensation Level
DSP	Digital Signal Processing
HA	Hearing Aid
HCPs	Hearing Care Professionals
HF	High-Frequency
HI	Hearing-Impaired
HL	Hearing Loss
NAL	National Acoustical Laboratories
NH	Normal-Hearing
NLFC	Non-Linear Frequency Compression
NR	Noise Reducers
OTC	Over-The-Counter
PRISMA	The Preferred Reporting Items for Systematic Reviews and Meta-Analyses
PROMs	Patient-Reported Outcome Measures
SADL	Satisfaction Amplification in Daily Life
SRT	Speech Reception Threshold
WDRC	Wide Dynamic Range Compression
WHO	World Health Organization
