# Supplementary material for: Factors in the Effective Use of Hearing Aids among Subjects with Age-Related Hearing Loss: A Systematic Review
Source: J Clin Med. 2024 Jul 10;13(14):4027. doi: 10.3390/jcm13144027 (PMC11277177; doi:10.3390/jcm13144027)
Supplement: Supplementary file 1 [file jcm-13-04027-s001.zip › Table S2_Search Terms.pdf]

Table S2: Search Strategy for PubMed database

(((((((((((((((((((middle age[MeSH Terms]) OR (aged, 80 and over[MeSH Terms])) OR (centenarian[MeSH Terms])) OR (nonagenarians[MeSH Terms])) OR (age-related hearing loss [Title/Abstract])) OR (aged[Title/Abstract])) OR (elderly[Title/Abstract])) OR (senior[Title/Abstract])) OR (older[Title/Abstract])) OR (oldest old[Title/Abstract])) OR (middle aged[Title/Abstract])) OR (centenarians[Title/Abstract])) OR (nonagenarians[Title/Abstract])) OR (octogenarians[Title/Abstract])) NOT (baby[Title/Abstract])) NOT (child[Title/Abstract])) NOT (children[Title/Abstract])) AND ((((((((hearing disorders[MeSH Terms]) OR (presbycusis[MeSH Terms])) OR (presbycusis[Title/Abstract])) OR (hearing loss[Title/Abstract])) OR (hearing impairment[Title/Abstract])) OR (hard of hearing[Title/Abstract])) OR (hearing disorders[Title/Abstract])) NOT (tinnitus[Title/Abstract]))) AND ((((((hearing aids[MeSH Terms]) OR (hearing aids[Title/Abstract])) OR (conventional hearing aid[Title/Abstract])) NOT (cochlear implant[MeSH Terms])) NOT (bone anchored implant[Title/Abstract])) NOT (cochlear implant[Title/Abstract]))) AND ((((((((patient satisfaction[MeSH Terms]) OR (patient health questionnaire[MeSH Terms])) OR (satisfaction[Title/Abstract])) OR (satisfaction survey[Title/Abstract])) OR (patient satisfaction[Title/Abstract])) OR (patient health questionnaire[Title/Abstract])) OR (hearing aid connectivity[Title/Abstract])) OR (non audiological factor[Title/Abstract]))) AND ((((((((((((((signal processing, computer assisted[MeSH Terms]) OR (audiological factors[Title/Abstract])) OR (noise reduction[Title/Abstract])) OR (compression[Title/Abstract])) OR (WDRC[Title/Abstract])) OR (digital signal processing[Title/Abstract])) OR (signal processing features[Title/Abstract])) OR (directional microphone[Title/Abstract])) OR (hearing aid fitting[Title/Abstract])) OR (hearing

aid approach[Title/Abstract])) OR (insertion gain[Title/Abstract])) OR (aided response[Title/Abstract])) OR (frequency discrimination[Title/Abstract])) OR (tuning curves[Title/Abstract]))
