# Supplementary material for: Factors in the Effective Use of Hearing Aids among Subjects with Age-Related Hearing Loss: A Systematic Review
Source: J Clin Med. 2024 Jul 10;13(14):4027. doi: 10.3390/jcm13144027 (PMC11277177; doi:10.3390/jcm13144027)
Supplement: Supplementary file 1 [file jcm-13-04027-s001.zip › Table S3_Study Characteristics and CCAT scores.pdf]

Author (Year), Country of Origin	Study Design	Participant Characteristics			HA owner experience		HA fitting			
		Sample size	Age (range| mean, years)	Gender	First Time User	Experienced	Instruments (surveys)	Speech-In-Quiet / Speech-In-Noise Test	Manufacturer(s)	Others
Abrams et al, (2012) USA	randomized crossover study	22	60-89 | 77.95	22 M		x	APHAB	_	Microtech, Phonak, Starkey, Siemens	_
Anderson et al. (2018) USA	randomized crossover study	49	54-90 | 72	19 F	x		SSQ. EAR	_	1 manufacturer (*NA*)	_
Banerjee (2011) USA	non-randomized crossover study	10	49-78 | 63,3	5 F | 4 M		x	satisfaction survey created on SnapSurvey	_	Starkey	multimemory (MM) et/ou volume control (VC) // + application
Bennett et al. (2018) Australia	cross-sectional crossover study	17 patients / 21 clinicians	67-88 | 75.94	11 F | 6 M		x	_	_	6 manufacturers (*NA*)	_
Bentler et al. (2008) USA	randomized crossover study	25	42- 79 | 65.1	12 F | 13 M	x	x	COSI, APHAB, GDS, Utley screening test, diary journal	I-SPIN, CST	Starkey	push button to access multiple programs
Bertozzo et al. (2019) Brasil	randomized crossover study	60	62 - 90| 77.52	24 F | 36 M	x		_	HINT	1 manufacturer (*NA)*	_
Blamey et al. (2006) Australia	non-randomized crossover study	8	32-82 | 69.6	3 F | 5 M		x	satisfaction survey ("comparative questionnaire"), SSQ	HINT	*NA*	push button to access multiple programs
Boymans et al. (2009) The Netherlands	retrospective study	689	mean 67	48% F | 52% M		x	AVETA, abbreviated version of HHDI, 5 subscale of AIHDH, AV subscale of APHAB, IOIHA	CVC words	*NA*	_
Campos et al. (2012) Brasil	randomized controlled trial	50	39-88	20 F | 30 M	x		IOI-HA	HINT	1 manufacturer (*NA*)	_
Chen et al. (2020) China	randomized crossover study	30	43-83 | 67.93	7 F | 23 M	x		rate loudness, clearness naturalness of sound quality	consonant recognition test, mandarin vowel list, tone recognition, HINT	Phonak	_
Cho et al. (2022) South Korea	retrospective study	1464	Mean 70.4	646 F | 818 M	*NA*	*NA*	K-HHIE, face-to-face survey (demographic, audiological, HA-related)	_	*NA*	_
Desjardins et al. (2009) USA	cross-sectional crossover study	50	46 - 89 | 75,36	23 F | 27 M		x	PHAST, APHAB, SADL + 22 questions on HA use and knowledge, demographic HA information	_	*NA*	_

Author (Year), Country of Origin	Study Design	Participant Characteristics			HA owner experience		HA fitting			
		Sample size	Age (range| mean, years)	Gender	First Time User	Experienced	Instruments (surveys)	Speech-In-Quiet / Speech-In-Noise Test	Manufacturer(s)	Others
DiGiovanni et al. (2010) USA	non-randomized controlled trial	20	exp 1 : 24-87 | 62.6 + 10 NE (exp 1-2)	exp 1 : 6F |4 M	*NA*	*NA*	_	_	exp 1 : Widex // exp 2 : Starkey	_
Dwarakanath et al. (2020) India	cohort study	42	60 - 78 | 71.6	15 F |19 M	x		ALHQ, EPI, IOI-HA	_	*NA*	_
Ferguson et al. (2016) UK	cohort study	30	52-88 | 68,4	12 F |18 M	x		MARS-HA, LQ1, LQ2, ECHO, GHAPB, SADL	_	Phonak Nathos Micro	_
Gatehouse et al. (2006a) UK	randomized crossover study	50	54-82 | 67.1	28 F |22 M	x		SADL, APHAB, GHAPB, HAPQ	_	Oticon	volume control only on NAL-RP fitting.
Gatehouse et al. (2006b) UK	randomized crossover study	50	54-82 | 67.1	28 F |22 M	x		cognitive abilities, self-reports (ALDQ)	_	Oticon	_
Hausladen et al. (2022) USA	non-randomized crossover study	24	48-81 | 61	5 F | 19 M	x		SSQ12, satisfaction ratings, ANL	QuickSIN, HINT	Unitron	_
Humes et al. (2009) USA	non-randomized controlled trial	213	74,6	34% F | 66% H	*NA*	*NA*	HAPI, GHAPB, MarkeTrak IV	_	*NA*	_
Humes et al. (2017) USA	randomized controlled trial	163	55-79 | 69,1	44% F | 56% M	x		PHAP, HHIE	CST	ReSound	_
Johnson et al. (2007) USA	non-randomized crossover study	16	58-84 | 75	*NA*	*NA*	*NA*	_	_	2 manufacturers (*NA*)	_
Karah et al. (2022) Israel	longitudinal study	53	40-60	*NA*	x	x	HHIE, COSI	detection, discrimination, identification, and comprehension quiet and noisy conditions	Hansaton	home-based auditory exercises via an online platform
Keidser et al. (2008) Australia	non-randomized crossover study	Exp 1 : 25 // Exp 2 : 12	Exp 1 : 18-87 | 75	Exp 1 : 3 F | 22 M		x	_	_	*NA*	_
Keidser et al. (2013) Australia	randomized crossover study	26	67-89 | 79	5 F | 21 M		x	_	_	Siemens	remote control (volume control)
Korhonen et al. (2013) Denmark	non-randomized crossover study	13	65 - 83 | 73	8 F | 5 M		x	_	ORCA-NST	Widex	_
Korhonen et al. (2017) Denmark	non-randomized crossover study	15	31 - 82 | 70.1	8F | 7 M		x	_	ORCA-NST	Widex	_
Laperuta et al. (2012)	cohort study	22	63 - 87|	11 F | 11 H	x		SADL	_	*NA*	_

Author (Year), Country of Origin	Study Design	Participant Characteristics			HA owner experience		HA fitting			
		Sample size	Age (range| mean, years)	Gender	First Time User	Experienced	Instruments (surveys)	Speech-In-Quiet / Speech-In-Noise Test	Manufacturer(s)	Others
Lelic et al. (2023) Denmark	randomized controlled trial	Ctrl grp : 10 // PF grp : 11	64 | 68	Ctrl grp : 3 F | 7 M // PF grp : 3 F | 8 M		x	BFIP, IOI-HA, HEARLI-Q, COSI	_	Widex	MyHearingExperience app
Mispagel et al. (2006) USA	non-randomized crossover study	10	mean 70,8	2F | 8 M		x	Listening Task Questionnaire, Environmental Sounds Questionnaire	HINT	*NA*	volume control (20dB range) + push button to access multiple programs (3)
Mondelli et al. (2012) Brasil	cohort study	30	mean 76,8	13 F | 17 M	x		WHOQOL	_	*NA*	_
Moore et al. (2010) UK	non-randomized crossover study	11	45 - 74 | 67	6 F | 5 M	x	x	PAL, APHAB, Overall sound quality rating	_	Starkey	_
Munro et al. (2005) UK	non-randomized crossover study	16	mean 70,8	8 F | 8 M	x		_	BKB	Phonak	_
Narayanan et al. (2021) India	cross-sectional crossover study	11	23-55 | 41,09	3 F | 8 M	x		_	Kannada paired-word list, Kannada word identification	*NA*	_
Naylor et al. (2015) Sweden	randomized crossover study	Exp 1 : 24 | Exp 2 : 16	Exp 1 : 72,3 |Exp 2 : 67,5	Exp 1 : 3 F | 21 M // Exp 2 : 5 F |11 M	Exp 2 x	Exp 1 x	HHIE, IOI-HA, HAPQ, short preference survey (which HA was prefered)	_	Oticon	_
Neher et al. (2016) Germany	randomized crossover study	60	60 - 82 | 72	*NA*		x	Reading Span, executive control, noise sensitivity, personality, physical test setup	_	Siemens	_
Oberg et al. (2007) Sweden	cross-sectional crossover study	162	36 - 80 | 66.6	66 F | 96 M	x		HHIE, SADL, CSS, IOI-HA, SOC, HADS	_	*NA*	_
Oberg et al. (2008) Sweden	randomized controlled trial	38	Grp 1 (trt) : 67.1 // Grp2 (ctrl) : 65.5	Grp1 : 7 F |12 M // Grp 2 : 6F | 13M	x		ECHO, SADL, HHIE, CSS, IOIHA, HADS, SOC, COSI. Speech recognition measurement.	_	Oticon	_
Oberg et al. (2014) Sweden	non-randomized crossover study	23	mean 87	11F | 12 M		x	HHIE, CSS, EuroQol 5D, GDS, IOI-AI, COSI, feedback survey (what they liked about the program)	_	*NA*	_

Author (Year), Country of Origin	Study Design	Participant Characteristics			HA owner experience		HA fitting			
		Sample size	Age (range| mean, years)	Gender	First Time User	Experienced	Instruments (surveys)	Speech-In-Quiet / Speech-In-Noise Test	Manufacturer(s)	Others
Palmer et al. (2006) USA	non-randomized controlled trial	49 HI | 30 NE	27-85 | 62.1 // mean 34.8 NE	HI : 22 F | 27 M // NE : 20 F |10 M	x	x	APHAB, IOI-HA	_	Siemens	_
Plyler et al. (2006) USA	non-randomized crossover study	30	32-85	*NA*		x	satisfaction with five point scale	_	Starkey,	push button to access multiple programs (4)
Plyler et al. (2006) USA	non-randomized crossover study	20 (11 grp A, 9 grp B)	Grp A : 48-85 | 64.8 // Grp B :18-82 | 58.7	*NA*	x		APHAB	CST, HINT	Starkey	_
Plyler et al. (2013) USA	non-randomized crossover study	14	66,4	5F | 9 M	x		ANL, APHAB, satisfaction rating (5pts scale), listener preference.	HINT, HFWL	Bernafon	_
Plyler et al. (2015) USA	non-randomized crossover study	20	69.7	7F | 13 M		x	satisfaction survey	nonsense VCV syllabe	Bernafon	_
Plyler et al. (2019) USA	non-randomized crossover study	15	55-83 | 73	2 F | 13 M		x	_	HFWL	Phonak	_
Recker et al. (2020) USA	non-randomized crossover study	20	30-84 | 64	*NA*		x	ANL	_	*NA*	_
Searchfield et al. (2018) New Zealand	non-randomized crossover study	25	mean 73	12 F | 13 M		x	HASP, SSQ12	HINT	Phonak	remote control (to access multiple programs)
Solheim et al. (2018) Australia	cross-sectional crossover study	181	mean 79.2	98 F | 83 M	x	x	questions about HA use (handling, sound quality, perceived need, benefit, earmold/dome, economic factor, functional factors, cosmetic factors, health-related factors)	_	*NA*	telecoil, streaming, bluetooth
Tye-Murray et al. (2022) USA	cross-sectional crossover study	30	60-86 | 72.3	13 F | 17 M	x		Satisfaction questionnaire	_	Intricon	DTx
Wu et al. (2010) USA	non-randomized controlled trial	24	36-79| 64	14 F |10 M	x	x	_	CST, HINT	Starkey	push button to access multiple programs
Wu et al (2019) USA	cross-sectional crossover study	54	65-88 | 73.6	28 F | 26 M	x	x	listening effort, sound quality, localization test, APHAB, SSQ, SADL	HINT	One manufacturer (*NA*)	EMA (Ecological Momentary Assessment) : collect real-time experience + smartphone

Wu et al (2020) USA	non-randomized crossover study	39	48 - 83 | 71	18 F | 21 M	x	x	HHIE, APHAB, SADL, SSQ, GHAPB	AFHAF	*NA*	EMA + smartphone
Wu et al (2019) China	non-randomized crossover study	73	60-95 | 77.1	27 F | 37 M	x		IOI-HA	_	*NA*	_
Yakunina et al (2021) Republic of Korea	randomized controlled trial	103	mean 53,6	18 % F | 80 % M	x		APHAB	_	Widex	_
Zakis et al (2012) Australia	non-randomized crossover study	12	20- 81	6 F | 6 M	*NA*	*NA*	_	_	Oticon	_

Notes : **ANL** : Acceptable Noise Level. **APHAB** : Abbreviate Profile of Hearing Aid Benefit. **AFHAF** : Four Alternative Auditory Feature test. **AIHDH** : Amsterdam Inventory for Hearing Disability and Handicap. **ALDQ** : Auditory Lifestyle and Demand Questionnaire. **ALHQ** : Attitude towards Loss of Hearing Questionnaire. **AVETA** : the Amsterdam Questionnaire for Unilateral or Bilateral Fitting. **BFIP** : Big Five Inventory Personality. **BKB** : Bamford Kowal Bench. **COSI** : the Client Oriented Scale of Improvement. **CSS** : the Communication Strategies Scale. **CST** : the Connected Speech Test. **EAR** : the Effectiveness of Auditory Rehabilitation. **ECHO** : Expected Consequeces of Hearing aid Ownership Questionnaire. **EMA** **:** Ecological Momentary Assessment. **EPI** : Eysenck Personality Inventory. **GDS** : Geriatric Depression Scale. **GHAPB** : Glagow Hearing Aid Benefit Profile. **HA** : Hearing Aid. **HADS** : the Hospital Anxiety and Depression Scale. **HAPI** : Hearing Aid Performance Inventory. **HAPQ** : Hearing Aid Performance Questionnaire. **HASP** : Hearing Aid Selection Profile. **HEARLI-Q** : Hearing-Related Lifestyle Questionnaire. **HFWL** : Pascoe's High Frequency Word List. **HHDI** : Hearing Handicap and Disability Inventory. **HHIE** : Handicap Inventory for the Elderly. **HINT** : Heaing in Noise Test. **IOI-AI :** the International Outcome Inventory - Alternative Interventions. **K-HHIE** : Korean version of the Hearing Handicap Inventory for the Elderly. **MARS-HA** : Measure of Audiologic Rehabilitation Self Efficacity for Hearing AIds. **MM** : MultiMemory. **NA** : Not Available Data. **NAL** : National Acoustical Laboratories. **ORCA-NST** : Office of Research in Clinical Amplification Nonsense Syllable Test. **PAL** : Profile of Aided Loudness. **PHAP** : Profile of Hearing Aid Performance. **PHAST** : Practical Hearing Aid Skills Test. **SADL** : Satisfaction with Amplification in Daily Life. **SOC** : the Sense of Coherence Scale. **SSQ** : Speech, Spatial and Qualities of Hearing Scale. **VC** : Volume Control. **WHOQOL** : World Health Organization Quality of Life Questionnaire.

Author (Year), Country of Origin	Study Description			Main Results	CCAT[a] Total Score[b]
	Aim	HA fitting methods	Duration		
Abrams et al, (2012) USA	To examine whether self-perception of hearing aid benefits differed as a function of hearing aid fitting method, specifically manufacturer's initial-fit approach versus a verified prescription.	NAL-NL1.	2 months (4-6 weeks for each condition)	Most participants preferred having their hearing aids programmed with the verified prescription. As hearing aid technology becomes more sophisticated, **clinicians may increasingly rely on the initial algorithm of manufacturers at the expense of verifying the hearing aid response using the current gold standard, probe-microphone measures.**	29
Anderson et al. (2018) USA	To determine how self-perceived performance varied as a function of modifications in signal processing using two self-report measures.	NAL-NL2	between 10 and 12 weeks	Results **support the use of self-report measures as effective tools in documenting the perceptual responses of listeners to hearing aid fittings** that vary substantially in the settings of the signal processing features.	29
Banerjee (2011) USA	To examine the real-world use of multimemory (MM) and volume (VC) controls and environmental characteristics that may influence their use.	eSTAT (Strakey's fitting)	4-5 weeks	The default setting acceptable 75% of the time. Volume control is preferred by patients and used in almost 10% of daily life situations. The findings of this study generally **support the use of synchronized MMs and/or VCs,** although there may be exceptions to their utility.	26
Bennett et al. (2018) Australia	To generate a conceptual framework for understanding hearing aid problems and how hearing aid owners respond to these problems and identify key aspects of the hearing aid fitting process that could be improved	*NA*	*NA*	Hearing aid management should be emphasized during clinical consultations to improve HA problems. Clinicians should **first identify the full range of problems** an individual is experiencing. P**roviding HA owners with information to prevent common problems during the initial appointment**. Deliver **training to clinicians** to improve their skills + ability to problems solve	29
Bentler et al. (2008) USA	To determine the impact of a digital noise reduction (DNR) scheme implemented in a current commercial hearing aid	NAL-NL1	5 months (3 to 4 weeks per condition)	Laboratory speech perception measures showed **no effect of the DNR**, with or without visual cues. Self-report measures indicated significantly **higher aversiveness in the DNR-off condition than pre-test scores.**	27
Bertozzo et al. (2019) Brasil	To comparatively analyze the NAL-NL2 and DSL v5.0a prescriptive methods according to the hearing aids individualized programming for the elderly with hearing loss.	NAL-NL2 or DSLv5.0	1h30 (one session)	**Equivalent performance between DSL v5.0a and NAL-NL2** prescription procedures in HA adaptation in the elderly with hearing loss. The amplification calculated by **DSL v5.0a provided better speech perception in silence.**	26
Blamey et al. (2006) Australia	To evaluate three different microphone configurations used together with the ADRO sound processing strategy.	*NA*	4-5 weeks	The **adaptive directional microphone has been shown to provide strong benefits in the majority of listening situations** (noise condition, preferred in 54% of situations).	26

Author (Year), Country of Origin	Study Description			Main Results	CCAT[a] Total Score[b]
	Aim	HA fitting methods	Duration		
Boymans et al. (2009) The Netherlands	To investigate the candidacy for bilateral versus unilateral fitting by combining a large number of case history and audiometric data coupled with fitting results and subjective data.	*NA*	*NA*	Better detection, reverberation and localization with bilateral HA vs unilateral. P**oorer for the comfort of loud sound for bila**t vs unilat. **Premium HA has higher scores for localization, speech in noise**, and less residual handicap than basic HA.	30
Campos et al.(2012) Brasil	To evaluate teleconsultation's efficacy for hearing aid fitting.	NAL-NL1	1 month	**Teleconsultation is an effective service model to perform hearing aid programming and verification** and to provide informational counseling and m**ay be used in situations where there is difficulty or an impediment for face to face procedures**	31
Chen et al. (2020) China	To examine the effects of NLFC fitting in hearing aids and auditory acclimatization on speech perception and sound-quality rating in hearing-impaired, native Mandarin-speaking adult listeners	Phonak Target (Phonak's fitting)	3-4 months	For native Mandarin-speaking listeners, the **NLFC** technology provided modest but significant i**mprovement in Mandarin fricative and sentence recognition**.	27
Cho et al. (2022) South Korea	To identify the differences in the characteristics of adopters and non-adopters of hearing aids (HAs); and to investigate factors influencing the purchase of HA.	*NA*	1 year, 2 months	Education level, duration of hearing loss, household income, and place of purchase were important factors associated with intention to purchase HA**, with third party reimbursement identified as the most prominent factor**. These findings suggest **that the government needs to play a more active role in increasing the distribution of HA to patients with hearing loss** in South Korea in the future.	35
Desjardins et al. (2009) USA	To assess experienced hearing aid users' ability to use their hearing aids correctly.	*NA*	2h (one session)	HA manipulation is an essential factor for HA success. Importance of directly assessing individual's ability to use their HA. The PHAST is an objective tool to evaluate HA users' ability to manipulate their HAs.	27
DiGiovanni et al. (2010) USA	To determine whether the responses obtained using the Widex Sensogram were equivalent to those obtained using current clinical threshold measurement methods. To assess the accuracy of the Starkey IREMS™ in measuring RECD values compared to a dedicated real-ear measurement system.	*NA*	exp 1 and 2: 1 session. Duration : NA.	**Real-ear measures are still required for verification of prescribed gain**, however, calling into question any claims of shortened fitting time. These technologies represent a positive direction in prescribing accurate gain during hearing-aid fittings, but **a stand-alone system is still the preferred method for real-ear measurements in hearing-aid fittings**.	25
Dwarakanath et al. (2020) India	To assess the influence of attitude towards loss of hearing and personality and the perceived hearing aid benefit in older adults.	NAL-NL1	2 months	Personality and attitude can cause variability in HA outcome measures. In**dividuals with extrovert personalities reaped better benefits when compared to individuals with an introverted personalities.** Individuals with a h**igher social and emotional impact of HL and awareness about HL performed better with HA.**	27

Author (Year), Country of Origin	Study Description			Main Results	CCAT[a] Total Score[b]
	Aim	HA fitting methods	Duration		
Ferguson et al. (2016) UK	To examine the impact of self-efficacy and expectations for hearing aids and readiness to improve hearing on hearing aid outcome measures in first-time adult hearing aid users.	NAL-NL 1	6 weeks	Self-efficacy for hearing aids does not appear to be a robust predictor of successful hearing aid outcomes using the measures in the present study. **MARS-HA is not the more appropriate survey to measure self-efficacity** for HA at the early stages of the fitting process. The assessment of the e**xpectation of HA and readiness to improve hearing may** help identify individuals attending audiology clinics.	32
Gatehouse et al. (2006a) UK	To evaluate the benefits of fast-acting WDRC, slow-acting AVC, and linear reference fittings for speech intelligibility and reported disability in a within-subject within-device masked crossover design on 50 listeners with SNHL.	NAL-RP	50 weeks (10weeks / fitting)	Each **nonlinear fitting was superio**r to the linear references f**or benefits in listening comfort, listener satisfaction, reported intelligibility and speech intelligibility**.	31
Gatehouse et al. (2006b) UK	To report measures of candidature from each of the above domains for a within-listener crossover design of linear and nonlinear amplitude compression systems with fast and slow time constants.	NAL-RP	50 weeks (10weeks / fitting)	B**etter performance with linear fitting**s is associated with **flatter audiograms, wider dynamic range**, and smaller differences in dynamic range between low and HF, and also with **more restricted auditory lifestyles**. **Better performance with all nonlinear fittings is associated with more sloping audiograms, more restricted dynamic ranges, greater differences in dynamic range between low and HF, and more varied auditory lifestyles.**	29
Hausladen et al. (2022) USA	To determine the effect of hearing aid technology level on listener outcome measures for new hearing aid users.	NAL-NL2	2x 4 weeks	Speech perception results showed no significant differences between technology levels on the HINT or QuickSIN.<br>Acceptable Noise Level **(ANL) was significantly improved for the premium devices**.<br>Subjective results showed significantly **improved satisfaction for speech in a large group and overall preference when using premium technology.**	32
Humes et al. (2009) USA	To compare differences in hearing-aid outcomes for groups of older adults fitted with different hearing-aid technologies, ranging from one-channel linear aids with output-limiting compression to four-channel wide-dynamic-range-compression devices with directional microphones.	*NA*	4-6weeks	Sup**erior speech recognition in babble for directional HA**. All four technologies demonstrate significant improvement in speech recognition in babble.	23

Author (Year), Country of Origin	Study Description			Main Results	CCAT[a] Total Score[b]
	Aim	HA fitting methods	Duration		
Humes et al. (2017) USA	To determine the efficacy of hearing aids in older adults using audiology best practices, to evaluate the effectiveness of an alternative over-the-counter (OTC) intervention, and to examine the influence of purchase price on outcomes for both service-delivery models.	NAL-NL2	6 weeks	Consumer **OTC model: only slightly poorer outcomes than the AB** model. The purchase price did not affect outcomes, but a high percentage of those who rejected HA paid the typical price.	32
Johnson et al. (2007) USA	To examine the effect of two commercially implemented continuous, FFT-phase cancellation FBR systems on perceived sound quality by listeners with sensorineural hearing impairments.	NAL-NL1	*NA*	Results support the activation of the FFT phase cancellation FBR system without concern for a noticeable degradation of sound quality	27
Karah et al. (2022) Israel	To evaluate the effectiveness of a home-based auditory exercises program on speech perception in noise and subjective hearing outcomes among hearing aid users and non-users.	NAL-NL2	3 weeks	Home-based auditory exercises can supplement the diagnostic evaluation. **New users performed significantly better than non-users in all speech in noise tasks**; however, compared to the experienced users, performance differences depended on task difficulty. The findings indicate that **HA users, even new users, had better perceptual performance than their peers who did not receive hearing aids.**	34
Keidser et al (2008) Australia	To investigate the effect of the baseline starting response on self-adjustments of gain in different frequency bands.	*NA*	1 session. Duration : NA.	The ability to **select an optimum gain** setting from two different baseline response clearl**y varied across listeners**, especially at high frequencies. The clinical implication is that s**elf-adjustments should begin from an appropriately prescribed starting response**	27
Keidser et al (2013) Australia	To investigate the efficacy and reliability of training an HA in everyday environments.	NAL-NL2	8 weeks	Of those who training was practical for 75 to 80% and tented to result in higher overall satisfaction with the device. **Training could be ineffective for a small proportion** of people and should be c**linically managed by scheduling a follow-up appointment with those who are interested in training their devices in their everyday environments.**	32
Korhonen et al (2013) Denmark	To evaluate the effect of a transient noise reduction (TNR) algorithm on listening comfort, speech intelligibility in quiet, and preferred wearer gain in the presence of transients.	NAL-NL1	*NA*	Participants reported gr**eater preference for the SS-on** than for SS-off usin**g listening comfort** as a criterion**. SS algorithm did not degrade the identification of speech i**ncluding stop consonants.	28
Korhonen et al (2017) Denmark	To evaluate the effect of a new wind noise attenuation (WNA) algorithm on subjective annoyance and speech recognition in the presence of wind.	*NA*	*NA*	**Reduce subjective annoyance for wind** and improve speech identification performance at a speed of 5m/sec.	30

Author (Year), Country of Origin	Study Description			Main Results	CCAT[a] Total Score[b]
	Aim	HA fitting methods	Duration		
Laperuta et al (2012)	To analyze the satisfaction of elderly individuals in the first six months of use of hearing aids granted by SUS.	*NA*	6 months	**Self-assessment questionnaires have been incorporated into routine clinical practice** and can be used to evaluate several aspects, such as satisfaction with HA. The average positive factors and overall results **obtained after the third month of use were higher than those obtained after the first month.** The means of n**egative factors obtained in the sixth month of use were higher than those of the first and third** months.	25
Lelic et al. (2023) Denmark	To investigate whether focusing on positive listening experiences in everyday life does indeed improve the perceived hearing aid benefit and satisfaction in experienced HA users.	manufacturer fitting	3 weeks	Results showed that **focusing on positive listening experiences improves the perceived hearing aid benefit and satisfaction in experienced hearing aid users.** This technique could be utilized in clinics to improve hearing aid users' experience with hearing aids.	27
Mispagel et al (2006) USA	To evaluate the effect of increasing the number of processing channels from 32- to 64-signal processing channels on subjects' loudness comfort and satisfaction, sentence recognition, and sound quality of their voice.	manufacturer fitting	11 weeks	**No difference between 32 to 64 channel conditions i**n loudness comfort or satisfaction, in sentence recognition in quiet or noise, and in sound quality of the subject's own voice.	26
Mondelli et al (2012) Brasil	To identify through the WHOQOL the quality of life of hearing-impaired individuals before and after hearing aid fittings.	*NA*	3 months	Significant improvement in quality of life in general. Necessary to cre**ate or implement programs to reintegrate the individual into society, especially the elderly.**	26
Moore et al (2010) UK	To describe an evaluation of the method, using a 16-channel behind-the-ear hearing aid incorporating slow-acting compression and providing gain for frequencies up to 7500 Hz.	*NA*	12 to 28 days	CAMEQ2-HF leads to reasonable loudness in a variety of everyday situations. Several participants noted that **transient sounds** were intrusive and/or **too loud.** This may **indicate a need to slightly reduce the gain prescribed by CAMEQ2HF when slow acing compression is used.**	26
Munro et al (2005) UK	To utilize sound quality judgments to compare two frequency responses in new users at 4-week intervals over a 24-week post-fitting period.	DSL	24 weeks	Subjects generally p**refer greater amplification at high frequencies for speech clarity**, but they **prefer reduced high-frequency amplification for comfort** and overall preference.	27
Narayanan et al. (2021) India	To evaluate the difference in real-ear aided response, real-ear insertion gain, aided thresholds, articulation index, and word recognition score in quiet with hearing aid programmed to NAL-NL1 first-fit and optimized-fit using probe-microphone verification	NAL-NL1	1 day	The **optimized-fit yields better audibility and improved word recognition in quiet**. The **probe-microphone** measurement is considered as the "**Gold Standard" for verification of hearing aid fitting** in the best practice guidelines for hearing aid fitting. That **lack of verification will result in under-amplification in the higher frequencies**, that in turn cause inaudibility for soft and average level sounds and this in turn would compromise the speech recognition.	27

Author (Year), Country of Origin	Study Description			Main Results	CCAT[a] Total Score[b]
	Aim	HA fitting methods	Duration		
Naylor et al (2015) Sweden	To test the idea that embodied narratives might affect outcomes in hearing aid fitting.	NAL-NL1	5 weeks	The **narrative embodied** in a given fitting process can substantially affect **the perceived benefit of the treatment**, independent of any acoustical differences, at **least for experienced users**. For **first-time users**, **acclimatization seems to overshadow the purely narrative effect of any fitting process.**	32
Neher et al (2016) Germany	To investigate if (1) equivalent links exist for different types of directional processing (DIR) and NR, (2) self-reported noise sensitivity and personality can account for additional variability in preferred DIR and NR settings, and (3) spatial target speech configuration interacts with individual DIR preference.	Connex (manufacturer's fitting)	3 sessions of 1h30	**PTA and executive control influence preference for DIR and NR settings**. Noise sensitivity and personality did not influence DIR and NR outcomes. **Higher PTA was associated with a stronger preference for bilateral DIR.**	28
Oberg et al (2007) Sweden	To collect descriptive data and to evaluate the psychometric properties of a range of self-report questionnaires in a Swedish population.	*NA*	1 year	P**sychosocial well-being** and **residual participation restriction factors are essential in hearing aid rehabilitation.** The number of questionnaires could be reduced and suggested using HADS, IOIHA, and CSS. **Assessment of psychosocial health before and after HA rehabilitation** can answer how much HA rehabilitation affects this variable. Importance **of subjective measurements**.	27
Oberg et al (2008) Sweden	To evaluate the effects of an individual pre-fitting intervention for first-time hearing aid users.	NAL-NL1	1 year	The fitting sound awareness failed to show any benefits over and above the effects of HA fitting.	33
Oberg et al (2014) Sweden	To explore the use of the Active Communication Education (ACE) program in an older-old population of people aged 87 yr in Sweden.	*NA*	5 weeks / 2h (ACE). 3 weeks post-program et 6 months post-program	**No significant differences pre and post ACE** on the outcome measures, but the **elderly found the program to be beneficial** and 90% stated that the course had increased their ability to deal with their HL and the problems it creates.	34
Palmer et al (2006) USA	To determine how a specific digital noise reduction system affects hearing aid users' perception of noise annoyance and aversiveness and compare their perceptions to those of normal-hearing listeners.	NAL-NL1	3 weeks	Perceived a**nnoyance and aversiveness increased with amplification**. Need for **counseling patients about realistic expectations related to annoyance** and aversiveness of sounds at the time of HA fitting.	25
Plyler et al (2006) USA	To investigate the subjective evaluation of expansion time constants in single-channel wide dynamic range compression (WDRC) hearing instruments.	NAL-NL1	2 weeks	**Fast-acting expansion did not significantly degrade satisfaction and was preferred overal**l by a significant number of hearing instrument users.	26

Author (Year), Country of Origin	Study Description			Main Results	CCAT[a] Total Score[b]
	Aim	HA fitting methods	Duration		
Plyler et al (2006) USA	To determine if amplifying beyond 2 kHz affected the objective and subjective performance of hearing instrument users with varying degrees of mild-to-severe high-frequency sensorineural hearing loss.	DSL io	12 weeks	**HF amplification significantly improved objective performance in noise** and subjective preference in quiet for listeners with varying degrees of mild-to-severe high-frequency hearing loss. D**ispensers should be aware that high-frequency amplification should initially be provided to the affected high-frequenc**y regions when mild-to-severe hearing loss is present.	24
Plyler et al (2013) USA	To determine the effects of multichannel wide dynamic range compression and ChannelFree processing on listeners' performance and/or preference using open canal hearing instruments.	NAL-NL1	10 weeks	Performance with **WDRC was not significantly different than performance with ChannelFree processing on any speech perception tas**k. Satisfaction ratings and preference results were not significantly different between the processing strategies.	30
Plyler et al (2015) USA	To determine whether CF processing provides equal or better consonant identification and subjective preference than WDRC.	NAL-NL1	1 session. Duration : NA	**Both signal (WDRC et CF)** processing strategies were effective and **provided comparable consonant identification improvement in quiet and in noise** over no amplification. Preference results are not significant between the processing strategies.	30
Plyler et al (2019) USA	To determine the effects of NLFC and DNR in noise on word recognition and satisfaction ratings in noise in adult hearing aid users.	DSL v5	1 session. Duration : NA	Activating NLFC or DNR in isolation or in combination did not significantly impact word recognition in noise. **Activating NLFC in isolation reduced satisfaction ratings relative to the DNR or combination condition (= NLFC+DNR)**.	30
Recker et al (2020) USA	To examine whether people with low and high acceptable noise levels (ANLs) have different preferences for the aggressiveness of noise reduction (NR) and microphone mode and whether they get various noise tolerance benefits with these two features.	NAL-NL2	5 weeks	Almost **all participants preferred the most aggressive NR and directional microphone** settings tested. **ANLs could not be used to prescribe these hearing aid settings differentially.**	29
Searchfield et al (2018) New Zealand	To compare preference for and performance of manually selected programs to an automatic sound classifier, the Phonak AutoSense OS.	NAL-NL2	8 weeks	S**RT performance was best** when a participant used programs for different environments based on **a sound classifier rather than manual selection.** Manual programs may still be valuable when customized for specific, atypical soundscapes, whereas c**ommon speech-in-noise situations may be better served by reliable automatic program** selection.	29
Solheim et al (2018) Australia	To describe older adults' experiences and issues with HAs in the first six months of use.	*NA*	6 months	The **most frequently reported issues were associated with handling, the earmold, and sound quality**, many of which can and should be easily addressed. A **follow-up support is needed to improve user experience among HA recipients.**	32

Author (Year), Country of Origin	Study Description			Main Results	CCAT[a] Total Score[b]
	Aim	HA fitting methods	Duration		
Tye-Murray et al. (2022) USA	To investigate whether a DTx could help older adults better adjust to their hearing loss and acclimate to new hearing aids.	NAL-R	8 weeks	Results **support the use of a hearing health care** DTx, paving the way for **audiologists to be able to more easily and efficiently incorporate follow-up aural rehabilitation into their routine** clinical services and to be able to provide services remotely. Many participants noted that the **daily curriculum provided useful information and tips for managing the listening environment** and listening challenges related to hearing loss.	27
Wu et al (2010) USA	To determine if older and younger adults can obtain and perceive comparable benefits afforded by DMHAs.	manufacturer fitting	4 weeks	Age **did not have a significant effect on directional benefit.** However, the field data showed that older age was significantly associated with a lower preference for the directional mode. **Audiologists should pay more attention to clients' social lifestyles before fitting** DMHAs. Providing a directional microphone may not be helpful for those who do not encounter many demanding listening environments.	25
Wu et al (2019) USA	To investigate the laboratory efficacy and real-world effectiveness of advanced directional microphones (DM) and digital noise reduction (NR) algorithms relative to basic-level DM/NR features of contemporary hearing aids.	NAL-NL2	20 weeks	Results of laboratory test: premium DM/NR effect on improving speech understanding and localization accuracy. However, both retrospective and in situ self-reports demonstrated that participants were more satisfied with HA when DM/NR features were turned on. No substantial evidence to support the effectiveness of premium HA in the real world. Older adults with mild to moderate HL are unlikely to perceive the additional benefits of premium DM/NR features in their daily lives.	34
Wu et al (2020) USA	To compare the sensitivity of in-situ and retrospective self-reports in detecting the outcome difference between hearing aid technologies and to determine the association between in-situ and retrospective self-reports.	NAL-NL2	8 weeks	HA2 is preferred over HA1. In situ self-report. EMA° could have a higher sensitivity than retrospective questionnaires. EMA is worth comparing different HA technologies. In situ et retrospective Q assess different aspects of HA outcome: w**eak association between two types of measures.**	29
Wu et al (2019) China	To investigate the satisfaction of age-related hearing loss or presbycusis patients with individual, accurate, and precise fitting progress, which is a priority for bilateral hearing aids, and to explore the related influencing factors and their role in predicting the efficiency of hearing aids.	*NA*	at least 3 months	Clients with bilateral HA have significantly higher scores compared with unilateral fittings. Age and first fitting are not meaningful to satisfaction with HA. A higher max speech recognition before HA could predict better efficiency and satisfaction with HA. Completing a speech recognition ability examination before fitting would make a significant contribution to the efficiency of hearing aids and help ARHL patients have realistic expectations.	32
Yakunina et al (2021) Republic of Korea	To compare LFT (linear frequency transposition) to routine wide dynamic range compression (WDRC) within the same open-fit HA device in demographically and audiologically matched subjects with HFHL.	NAL-NL1 and NAL-NL2.	3 months	**LFT not provide an additional benefit** for subjects with HFHL. Users p**referred conventional Has featuring WDRC**.	33
Zakis et al (2012) Australia	To investigate preferences between low delays and phase-frequency responses of behind-the-ear, open-canal hearing aids with acoustic conditions deemed sensitive to delay effects by normal-hearing listeners.	*NA*	2h (one session)	Preferences between all other processing conditions were not significant. **Whether different phase-frequency responses may be preferred with different music stimuli is unclear.**	34

Notes : **ANL** : Acceptable Noise Level. **APHAB** : Abbreviate Profile of Hearing Aid Benefit. **ADRO** : Adaptative Dynamic Range Optimization, **DM** : Directional Microphones. **CF** : ChannelFree. **CSS** : the Communication Strategies Scale. **DIR** : Directional Processing. **DMHA** : Directional Microphone Hearing Aid. **DNR** : Digital Noise Reduction. **DSL** : Desired Sensation Level. **EMA :** Ecological Momentary Assessment. **HA** : Hearing Aid. **HADS** : the Hospital Anxiety and Depression Scale. **HFHL** : High Frequency Hearing Loss. **HINT** : Heaing in Noise Test. **HL** : Hearing Loss. **IOI-AI :** the International Outcome Inventory - Alternative Interventions. **LFT** : Linear Frequency Transposition. **MARS-HA** : Measure of Audiologic Rehabilitation Self Efficacity for Hearing AIds. **MM** : MultiMemory. **NA** : Not Available Data. **NAL** : National Acoustical Laboratories. **NLFC** : nonlinear frequency compression. **NR** : Noise Reduction. **NST** : Office of Research in Clinical Amplification Nonsense Syllable Test. **PHAST** : Practical Hearing Aid Skills Test. **TNR** : transient Noise Reduction. **VC** : Volume Control. **WDRC** : Wide Dynamic Range Compression. **WHOQOL** : World Health Organization Quality of Life Questionnaire. **WNA** : Wind Noise Attenuation.

[a]**CCAT**: Crowe Critical Appraisal Tool.
[b]**Total CCAT** score is 40 points.

Table S3 : Characteristics of Included Studies and Total Score for Crowe Critical Appraisal Tool.
