# Supplementary material for: Factors in the Effective Use of Hearing Aids among Subjects with Age-Related Hearing Loss: A Systematic Review
Source: J Clin Med. 2024 Jul 10;13(14):4027. doi: 10.3390/jcm13144027 (PMC11277177; doi:10.3390/jcm13144027)
Supplement: Supplementary file 1 [file jcm-13-04027-s001.zip › Table S4_Category-wise and Total Critical Appraisal Scores for the 54 Studies Included in the Review.pdf]

Author (Year), Country of Origin	Preliminaries	Introduction	Design	Sampling*<br>2. Participants/cases/groups: inclusion ❑ and exclusion ❑ criteria		Data collection	Ethical matters	Results	Discussion	Total score
Abrams et al. (2012) USA	4	5	4	*1*	4	3	2	3	4	**29**
Anderson et al. (2018) USA	4	5	4	*1*	3	3	3	3	4	**29**
Banerjee (2011) USA	4	5	3	*1*	3	3	2	2	4	**26**
Bennett et al. (2018) Australia	4	5	4	*1*	2	3	3	3	5	**29**
Bentler et al. (2008) USA	4	5	4	*2*	3	3	2	3	3	**27**
Bertozzo et al. (2019) Brasil	3	5	3	*1*	2	3	3	3	4	**26**
Blamey et al. (2006) Australia	4	5	3	*1*	2	3	1	4	4	**26**
Boymans et al. (2009) The Netherlands	4	5	4	*2*	4	3	1	4	5	**30**
Campos et al. (2012) Brasil	4	5	4	*1*	4	3	2	4	5	**31**
Chen et al. (2020) China	4	5	3	*1*	2	3	3	3	4	**27**
Cho et al. (2022) South Korea	4	5	4	*1*	4	5	3	5	5	**35**
Desjardins et al. (2009) USA	4	5	3	*1*	3	3	1	3	5	**27**
DiGiovanni et al. (2010) USA	3	5	3	*1*	2	3	2	3	4	**25**
Dwarakanath et al. (2020) India	4	5	3	*1*	2	3	3	3	4	**27**
Ferguson et al. (2016) UK	3	5	4	*2*	5	5	3	3	4	**32**
Gatehouse et al. (2006a) UK	4	5	4	*2*	3	4	2	4	5	**31**
Gatehouse et al. (2006b) UK	4	5	4	*1*	2	3	2	4	5	**29**
Hausladen et al. (2022) USA	4	5	4	*1*	4	4	2	4	5	**32**
Humes et al. (2009) USA	4	5	3	*0*	2	3	1	3	2	**23**
Humes et al. (2017) USA	4	5	4	*2*	4	4	2	4	5	**32**
Johnson et al. (2007) USA	4	5	4	*0*	2	3	1	4	4	**27**
Karah et al. (2022) Israel	4	5	5	*2*	4	5	3	3	5	**34**

Author (Year), Country of Origin	Preliminaries	Introduction	Design	Sampling*		Data collection	Ethical matters	Results	Discussion	Total score
				2. Participants/cases/groups: inclusion and exclusion criteria						
Keidser et al. (2008) Australia	4	5	3	*1*	2	3	1	4	5	**27**
Keidser et al. (2013) Australia	3	4	4	*2*	4	4	3	5	5	**32**
Korhonen et al. (2013) Denmark	4	4	3	*1*	5	3	3	3	3	**28**
Korhonen et al. (2017) Denmark	4	5	3	*1*	5	3	3	2	5	**30**
Laperuta et al. (2012)	3	3	3	*1*	4	3	3	3	3	**25**
Lelic et al. (2023) Denmark	3	5	4	*2*	4	3	2	3	3	**27**
Mispagel et al. (2006) USA	4	5	3	*2*	2	3	3	2	4	**26**
Mondelli et al. (2012) Brasil	4	4	3	*2*	5	3	3	2	2	**26**
Moore et al. (2010) UK	4	4	3	*2*	3	3	2	3	4	**26**
Munro et al. (2005) UK	4	4	3	*2*	4	3	3	2	4	**27**
Narayanan et al. (2021) India	4	5	4	*2*	4	2	2	2	4	**27**
Naylor et al. (2015) Sweden	4	4	4	*2*	5	3	3	4	5	**32**
Neher et al. (2016) Germany	4	5	3	*2*	4	3	3	3	3	**28**
Oberg et al. (2007) Sweden	3	5	2	*1*	4	3	2	3	5	**27**
Oberg et al. (2008) Sweden	4	5	4	*2*	4	5	2	5	4	**33**
Oberg et al. (2014) Sweden	4	5	4	*1*	5	5	2	4	5	**34**
Palmer et al. (2006) USA	3	5	3	*1*	4	3	1	2	4	**25**
Plyler et al. (2006) USA	4	5	3	*2*	4	2	1	3	4	**26**
Plyler et al. (2006) USA	4	5	3	*2*	2	2	1	3	4	**24**
Plyler et al. (2013) USA	4	5	4	*1*	3	2	2	5	5	**30**
Plyler et al. (2015) USA	4	5	4	*1*	4	2	1	5	5	**30**

Author (Year), Country of Origin	Preliminaries	Introduction	Design	Sampling*		Data collection	Ethical matters	Results	Discussion	Total score
				2. Participants/cases/groups: inclusion ❑ and exclusion ❑ criteria						
Plyler et al. (2019) USA	4	5	4	*1*	4	2	1	5	5	**30**
Recker et al. (2020) USA	4	5	3	*1*	3	2	3	4	5	**29**
Searchfield et al. (2018) New Zealand	4	5	4	*1*	3	3	3	4	3	**29**
Solheim et al. (2018) Australia	3	5	3	*1*	4	5	3	4	5	**32**
Tye-Murray et al. (2022) USA	4	5	3	*1*	3	4	2	2	4	**27**
Wu et al. (2010) USA	4	5	2	*1*	3	2	0	4	5	**25**
Wu et al. (2019) USA	4	5	4	*1*	5	4	2	5	5	**34**
Wu et al. (2020) USA	4	5	2	*1*	5	2	1	5	5	**29**
Wu et al. (2019) China	4	5	4	*2*	4	5	2	4	4	**32**
Yakunina et al. (2021) Republic of Korea	4	5	5	*2*	4	5	4	4	2	**33**
Zakis et al. (2012) Australia	4	4	5	*2*	4	4	3	5	5	**34**

**Sampling : For the sampling category, we added a subsection detailing the inclusion and exclusion criteria to provide more detailed information for each study.*

Table S4 : Category-wise and Total Critical Appraisal Scores for the 54 Studies Included in the Review
