# Supplementary material for: Factors in the Effective Use of Hearing Aids among Subjects with Age-Related Hearing Loss: A Systematic Review
Source: J Clin Med. 2024 Jul 10;13(14):4027. doi: 10.3390/jcm13144027 (PMC11277177; doi:10.3390/jcm13144027)
Supplement: Supplementary file 1 [file jcm-13-04027-s001.zip › Table S5_Table of Normalized SRT Values and Subjective Satisfaction Scores Across Studies..pdf]

Authors	SIB50_norm	SIB50_paper	diff_x*	SIB50_sd*_paper	Satisfaction	Satisfaction_sd	Category*	Condition	Cohort	CCAT (Final score)
Blamey et al. (2006)	-2,6	-4,5	-1,9	0,5	54	*NA*	3	ADM*	8	26
	-2,6	2,6	5,2	0,6	17	*NA*	3	omnidirectional	8	
Campos et al. (2012)	-4,6	4,94	9,54	3,16	82	*NA*	2	control group	25	31
	-4,6	4,17	8,77	4,15	82	*NA*	2	experimental group (teleconsultation)	25	
Chen et al. (2020)	-4,3	-0,45	3,85	*NA*	85	*NA*	4	NLFC off	30	27
	-4,3	-0,12	4,18	*NA*	87	*NA*	4	NLFC on	30	
Mispagel et al. (2006)	-2,6	3,6	6,2	*NA*	54	17	2	32 channel	10	26
	-2,6	3,5	6,1	*NA*	59	22	2	64 channel	10	
Plyler et al. (2006)	-2,6	0,5	3,1	1	68	*NA*	2	max HF (group B*)	9	26
	-2,6	-0,3	2,3	1	76	*NA*	2	max HF(group A*)	11	
	-2,6	1	3,6	0,6	68	*NA*	2	min HF (group A)	11	
	-2,6	2,8	5,4	1,2	67	*NA*	2	min HF(group B)	9	
Plyler et al. (2013)	-2,6	-1,5	1,1	2	78	14	4	CF*	14	30
	-2,6	-1	1,6	3	42	12	4	unaided	14	
	-2,6	-2	0,6	2,5	76	11	4	WDRC*	14	
Wu et al. (2020)	-2,6	1	3,6	2	72	1	1	basic off	54	34
	-2,6	-1	1,6	2	76	1	1	basic on	54	
	-2,6	1	3,6	2	74	1	1	premium off	54	
	-2,6	-2	0,6	2	76	1	1	premium on	54	
Hausladen et al. (2022)	-2,6	3,1	5,7	3,6	82	18	2	premium	24	32
	-2,6	4,9	7,5	3,1	74	25	2	basic	24	

**diff_x=SIB50paper-SIB50_norm. Sd=standard deviation. Category 1 = noise reduction ; 2 = fitting ; 3 = microphone ; 4=compression. ADM = Adaptative Directional Microphones. Group A = high-frequency pure-tone average (3.4.6kHz) ≤ 55dB HL. Group B = 56dB HL ≤ high-frequency pure-tone average ≤ 75dB HL. CF = ChannelFree. WDRC = Wide Dynamic Range Compression.*

Table S5 : Table of Normalized SRT Values and Subjective Satisfaction Scores Across Studies.
